# A randomized, double‐blind, placebo‐controlled, dose‐escalating phase IIa trial to evaluate the safety, tolerability, efficacy, and pharmacokinetics of multiple oral doses of Pynegabine tablets as add‐on therapy in patients with focal epilepsy

**DOI:** 10.1111/cns.70002

**Published:** 2024-09-09

**Authors:** Shan Wei, Weng Shiwen, Chang Cao‐wenjing, Yang Huajun, Wang Qun

**Affiliations:** ^1^ Department of Neurology, Beijing Tiantan Hospital Capital Medical University Beijing China; ^2^ China National Clinical Research Center for Neurological Diseases Beijing China; ^3^ Beijing Institute for Brain Disorders Beijing China; ^4^ Sanbo Brain Hospital Capital Medical University Beijing China

**Keywords:** anti‐seizure medications, clinical trial, protocol, Pynegabin

## Abstract

**Aims:**

This study aims to investigate the safety, tolerability, efficacy, and pharmacokinetics of Pynegabine as an add‐on therapy in the treatment of focal epilepsy.

**Methodology:**

This is a protocol phase‐IIa, randomized, double‐blinded, placebo‐controlled, multicenter study in patients with focal epilepsy from multiple centers in China who have been treated with at least 2 ASMs without effective control. The study involves an 8‐week run‐in period with stable use of previous medications. Patients are then randomized to receive either Pynegabine or a placebo. Sentinel administration is performed initially, and subsequent patients are randomized based on safety assessments. Three dose cohorts (15, 20, and 25 mg/d) are established. Efficacy is assessed through various measures, including seizure frequency, CGI score, PGI score, HAMA score, HAMD score, MoCA scale score, QOLIE‐31 scale score, and 12 h‐EEG score. Safety evaluations, PK blood samples, concomitant medications, and adverse events are also recorded.

**Conclusion:**

Data from the study will be used to evaluate the safety, tolerability, efficacy, and pharmacokinetics of Pynegabine tablets as add‐on therapy for focal epilepsy.

## Graphical abstract

The protocol of exploring the safety, tolerability, effectiveness, and pharmacokinetic characteristics of Pyrenegabine tablets as an adjunctive treatment for focal epilepsy.

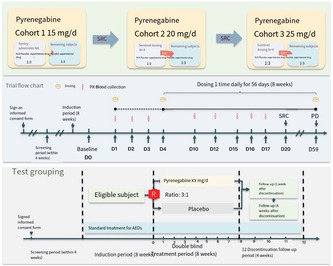

## 1 INTRODUCTION

Anti‐seizure medications (ASMs) reduce excitability of neuronal membranes by interacting with ion channels (e.g., sodium or calcium channels) or neurotransmitter receptor complexes (most promote inhibitory gamma‐aminobutyric acid (GABA) or interfere with the glutamate neurotransmission). Traditional ASMs used to treat focal epilepsy, such as phenytoin, carbamazepine, phenobarbital and valproate, although generally effective, tend to develop significant safety concerns (e.g. severe rash, osteoporosis, teratogenicity, liver failure) and pharmacokinetic issues. Newer antiseizure medications (ASMs) such as levetiracetam, lamotrigine, lacosamide, etc., while not showing significant efficacy advantages over traditional ASMs, are suggested to offer improved safety profiles for epilepsy patients. However, some adverse reactions are still unavoidable, among which neuropsychiatric symptoms such as fatigue, dizziness, instability, and irritability are the most common side effects.

The KCNQ potassium channel is an effective target for developing new ASMs, and its agonists are considered effective for epilepsy. In 2011, GlaxoSmithKline developed the first KCNQ potassium channel agonist, Retigabine, to be marketed. The primary pharmacological action of Retigabine is associated with voltage‐gated potassium channels (KCNQs), where modulation of M‐type potassium currents by acting on KCNQ2/3 channels is its primary mechanism of action, resulting in a hyperpolarized shift of potassium currents and a decrease in neuronal excitability. The anti‐seizure mechanism of Retigabine differs from other ASMs in that it does not act on NMDA or inward potassium currents in rat cortical neurons to weakly block neuronal sodium and mixed calcium currents but rather activates GABA‐induced currents. Due to its electron‐rich triamino structure on the benzene ring, Retigabine exhibits chemical instability, making it prone to oxidative degradation and darkening during synthesis and storage. Its severe side effect, retinal pigmentation, is also related to this instability. Additionally, it has poor metabolic properties and low distribution rates in the brain, resulting in suboptimal effectiveness as a central nervous system drug. Due to these issues and poor commercial prospects, GlaxoSmithKline no longer provides Retigabine to the market after June 2017.

Pynegabine is a new potent agonist targeting KCNQ potassium channels obtained by researchers from the Shanghai Institute of Materia Medica, Chinese Academy of Sciences, after in‐depth and systematic research on the structure–effect relationship and several rounds of structural optimization to modify the inherent structural deficiencies of the previously marketed anti‐seizure medication, Retigabine. Pynegabine is highly effective in stimulating KCNQ2‐5 channels, which are mainly expressed in the nervous system, but not the KCNQ1 channel, which is mainly expressed in the heart. Pynegabine inhibits spontaneous and evoked discharges of hippocampal pyramidal neurons and reduces neuroexcitability, resulting in anti‐seizure effects.

Pynegabine is indicated for refractory epilepsy in the form of tablets. On October 12, 2018, the Shanghai Institute of Materia Medica, Chinese Academy of Sciences obtained the clinical trial approval for Pynegabine Tablets, which belong to Class 1 chemical drugs according to the latest registration classification. The detail is elaborated on the methodology of the clinical trial, with the expectation of stimulating widespread discussion on the effectiveness of the experimental design. Based on the above introduction, this study was designed as a randomized, double‐blind, placebo‐controlled, multiple‐dose, dose‐escalation study to investigate the safety, tolerability, efficacy, and pharmacokinetics of Pynegabine tablets after multiple oral doses as add‐on therapy in patients with focal epilepsy.

The Pyrenegabine's Phase II study protocol serves as the initial step in evaluating the therapeutic potential of a drug candidate in a relatively small group of patients or study participants. These trials are primarily focused on assessing the drug's pharmacokinetics, pharmacodynamics, and preliminary efficacy, providing crucial insights that guide the decision‐making process for further development. We would like to understand Pyrenegabine's mechanism of action and its effects on the target patient population. By conducting these initial studies in a controlled setting, researchers can gather valuable data on the drug's absorption, distribution, metabolism, and elimination (ADME) characteristics and its impact on relevant biomarkers and clinical outcomes. Additionally, we would like to know the optimal dosing regimen and the appropriate patient subgroups most likely to benefit from the investigational therapy. This information is essential for designing and powering subsequent Phase III trials, which are larger in scale and focused on evaluating the drug's efficacy and safety more definitively.

## 2 METHODOLOGIES

### 2.1 Ethics and communication

The study will be conducted at multiple sites in China and will follow the guidelines for human trials summarized in the latest version of the Declaration of Helsinki. The protocol has been approved by the ethics committee (EC) of Capital Medical University affiliated Beijing Tiantan Hospital as the lead institution (Review number: YW2023–042‐01). Regular meetings will be held to monitor the progress of the study. After the study was completed, the results will be published in medical journals. Subject names will not appear in any published articles, and no data will be disclosed to anyone other than investigators and hospital EC members.

### 2.2 Research programme

This is a multicenter, randomized, double‐blind, placebo‐controlled, dose‐escalation clinical trial. This study will include patients with focal epilepsy who have been treated with at least 2 ASMs at therapeutic doses (a dose greater than 50% of the recommended daily dose of the ASM) without effective control at multiple centers in China. After the introductory period, eligible patients will be randomized into the trial group (Pynegabine) and the placebo group (3:1). Sentinel dosing will be conducted initially, followed by dosing of 4 randomized patients for assessment of serious adverse reactions or adverse events, then subsequent dosing of 8 randomized patients. A total of 3 dose cohorts will be set, and each dose cohort will enter the next dose cohort after completion of evaluation. The drug will be discontinued after 8 weeks of administration and patients will be continuously observed until 4 weeks after discontinuation. The seizure frequency (including the frequency of each subtype: focal awareness seizures, focal awareness‐impaired seizures, and focal onset bilateral tonic–clonic seizures), CGI score, PGI score, HAMA score, HAMD score, MoCA scale score, QOLIE‐31 scale score, NHS3 scale score and 12 h‐EEG score will be analyzed for comparison of efficacy. PK blood samples will be collected for the analysis of pharmacokinetic characteristics. Safety evaluation indicators will be observed, and concomitant drugs and adverse events will be recorded to evaluate the safety. The key assessments and evaluations to be performed at each visit are summarized in Table 1.

**TABLE 1:** Key assessments and evaluations at each visit.

Process	Time point											
	V1	Introductory period	V2	V3	V4	V5	V6	V7	V8	V9	V10	V11
Informed Consent Form	X											
Demographic information	X											
Medical history	X											
Randomization	X											
Trial drugs				X	X	X	X	X	X	X		
Physical examination	X		X				X	X			X	X
Vital signs	X		X	X	X	X	X	X		X	X	X
Height	X											
Body weight	X		X		X		X	X		X	X	X
12 h‐EEG	X		X				X			X		
Brain CT/MR (optional)	X		X									
PK blood sampling				X		X				X		
Blood routine	X		X		X		X	X		X	X	X
Blood biochemistry	X		X		X		X	X		X	X	X
Coagulation function	X		X		X		X	X		X	X	X
Urine routine	X				X		X	X		X	X	X
Urine toxicology test	X		X									
Residual urine volume after urination			X				X			X		X
Infectious disease screening	X											
Pregnancy test(optional)	X		X									X
OCT test	X						X			X		X
ECG test	X		X		X		X	X		X	X	X
CGI, PGI, C‐SSRS, HAMA, HAMD, MoCA scale, QOLIE‐31, NHS3	X (C‐SSRS)		X				X	X		X		
Medications/non‐drug therapies	X	X	X	X	X	X	X	X	X	X	X	X
Adverse events	X	X	X	X	X	X	X	;X	X	X	X	X

*Note*: X, result need to be collected; V1, Screening period: No more than 28 days; V2, Introductory period: 8 weeks +24 days (minimum 8 weeks, maximum 80 days); V3, Day 1 of administration; V4, Day 4 of administration; V5, Day 5 to Day 16 of administration; V6, Day 17 of administration; V7, Day 31 of administration; V8, Day 45 of administration; V9, Day 59 of administration; V10, Day 7 post‐discontinuation; V11, Day 28 post‐discontinuation.

Abbreviations: CGI, Clinical Global Impression; C‐SSRS, Columbia‐Suicide Severity Rating Scale; CT, Computed Tomography; ECG, Electrocardiogram; EEG, Electroencephalography; HAMA, Hamilton Anxiety Rating Scale; HAMD, Hamilton Depression Rating Scale; MoCA, Montreal Cognitive Assessment; MR, Magnetic Resonance Imaging; NHS3, National Hospital Seizure Severity Scale; OCT, Optical Coherence Tomography; PGI, Patient Global Impression; PK blood sampling, pharmacokinetic blood sampling; QOLIE‐31, Quality of Life in Epilepsy‐31.

### 2.3 Research patients

Patients with focal epilepsy who have been on stable use of 1–2 ASMs for at least 4 weeks are recommended to be enrolled in this study.

### 2.4 Inclusion criteria

(1) Age ≥ 18 years old and ≤ 75 years old; (2) patients diagnosed with focal epilepsy according to the investigator's clinical experience, the subject's medical history, EEG, neuroimaging and meeting the criteria of focal seizure and focal progression to bilateral tonic–clonic seizure in the seizure classification formulated by the International League Against Epilepsy (ILAE) 2017, that the seizure originating from the neural network within one hemisphere, it can start in a scattered manner from one or multiple locations, or even from wide‐spread origins, and it can also originate from subcortical structures; (3) during the 8‐week introductory period, subject seizure frequency needs to be ≥4 seizures per 28 days and the seizure‐free duration should not exceed 21 days; (4) the subject has used at least 2 ASMs (combined or sequential) at therapeutic doses (a dose >50% of the recommended daily dose of the ASM), not including the ASMs was discontinued due to side effects, before signing the informed consent form, but failed to effectively control the condition; (5) subjects has been on stable use of 1–2 ASMs for at least 4 weeks prior to informed consent, and is expected to remain stable during the 8‐week introductory period and the 8‐week treatment period; (6) subjects of childbearing potential are required to voluntarily take effective contraceptive measures after entering the screening period until 3 months after the completion of the trial; and (7) subjects voluntarily signed the informed consent form and voluntarily willing to cooperate with providing biological samples for testing and correctly completing diary cards as required by the protocol.

### 2.5 Exclusion criteria

(1) known allergies to any component of the investigated product; (2) non‐epileptic seizures (e.g. psychogenic non‐epileptic seizures); (3) only non‐motor focal seizures (ILAE, 2017 classification); (4) patients with treatable epilepsy etiology; (5) patients with current or past seizures too frequent to be accurately counted; (6) patients with a history of epilepsy cluster seizures within 3 months prior to signing the informed consent form; (7) patients with progressive intracranial exacerbation; (8) patients that have had epilepsy surgery within 8 months prior to signing the informed consent form; (9) patients with a history of cerebrovascular accident within 6 months prior to signing the informed consent form; (10) patients with macular edema or a history of retinopathy; (11) patients with serious uncontrolled diseases, such as cardiovascular disease, liver disease, kidney disease, acute infection, advanced tumor, etc.; (12) patients with serious heart disease; (13) patients with screening and baseline laboratory results meeting the following requirements: (ALT, AST, TBIL, Cr ≥1.5 times the upper limit of normal; ALP ≥2 times the upper limit of normal; creatinine clearance (CCr) < 60 mL/min; platelet count <80 × 109/L; neutrophil count <1.8 × 109/L; HBsAg antigen (or nucleic acid test) positive or HCV antibody (or nucleic acid test) positive or HIV antibody (or nucleic acid test) positive or Treponema pallidum antibody positive.); (14) QTcF interval significantly prolonged; (15) subjects who have received aminophenol within 24 weeks prior to signing the informed consent form, confirmed by visual field test to have aminophenol related abnormalities, and judged by the investigator to be still clinically significant; (16) subjects with severe drug hypersensitivity reactions to current ASMs or drug‐related rashes requiring hospitalization; (17) alcoholics and/or psychoactive substance and other drug abusers (Alcohol consumption: > 21 units per week for men and 14 units for women (1 unit =360 mL beer;150 mL wine; 45 mL spirits)); (18) termination of pregnancy <90 days prior to signing informed consent; pregnant and lactating women (currently nursing or not nursing but <6 months after delivery); (19) patients with obvious mental disorders; patients with behavioral or cognitive incapacity due to other reasons, including patients who are considered by the investigator to have poor compliance, which is likely to result in failure to complete the treatment course and follow‐up specified in the protocol; (20) previous suicidal behavior (including active attempts, interrupted attempts, or attempted attempts) or suicidal ideation within 6 months prior to screening (presumably by a positive response (“Yes”) to question 4 or 5 of the C‐SSRS at screening); and (21) has used the investigated drug/investigated medical device within 90 days prior to signing informed consent, or plans to participate in other clinical trials during this trial.

### 2.6 Research grouping

Trial group/placebo group.

Stable use of 1–2 ASMs for at least 12 weeks.

Eligible patients will be randomized into the trial or placebo group.

The dose was administered according to the preset dose of each cohort. The dose was administered once on the first day (D1), suspended for 2 days (D2 and D3); the dose was administered again on the fourth day (D4), once daily, for 8 consecutive weeks (D59). The dose was administered from D18 to D59. The dosing is suspended for 4 weeks.

### 2.7 Research process

#### 2.7.1 Screening period (V1): No more than 28 days

Prior to the study, medical history and demographic information will be obtained. Body weight, vital signs, physical examination, 12 h‐EEG, brain CT/MRI (optional), blood chemistry, blood routine, coagulation function, urinalysis, urine toxicology test, infectious disease screening, pregnancy test (optional), OCT, ECG will be performed. C‐SSRS (baseline survey/screening version) assessment will be performed. Diary cards will be issued and concomitant medications/non‐drug therapies and adverse events will be recorded.

Introductory period: 8 weeks +24 days (minimum 8 weeks, maximum 80 days).

Concomitant medications/non‐drug therapies, adverse events will be recorded.

Baseline (V2): Day −2 to Day 0 (D0 is defined as the randomization day).

Body weight, vital signs, physical examination, blood biochemistry, blood routine, coagulation function, urine routine, residual urine volume after urination, urinalysis, pregnancy test (optional), ECG examination will be performed. CGI, PGI, C‐SSRS (version since last visit), HAMA, HAMD, MoCA scale and QOLIE‐31 scale will be evaluated. Diary cards will be issued and recovered, and concomitant drugs/non‐drug therapies and adverse events will be recorded. Randomization will be conducted after review of entry and exit conditions.

#### 2.7.2 Treatment period (V3–V9): first dose to D59

Day 1 of administration (V3): Vital signs measurements, PK blood sampling, dispensing of trial medication, and recording of concomitant medications/non‐drug therapies, adverse events will be recorded.

Day 4 of administration (V4): Body weight, vital signs, blood biochemistry, blood routine, coagulation function, urine routine and ECG will be examined. Trial drugs will be dispensed, and concomitant drugs/non‐drug therapies and adverse events will be recorded.

Day 5 to Day 16 of administration (V5): Vital signs will be measured (only on Days 5, 6, 7, and 12 after administration), PK blood will be drawn, trial drug will be dispensed, and concomitant/non‐drug therapies and adverse events will be recorded.

On Day 17 of administration (V6): Body weight, vital signs, physical examination, 12 h‐EEG, blood biochemistry, blood routine, coagulation function, urine routine, residual urine volume after urination, OCT examination, ECG examination will be performed. CGI, PGI, C‐SSRS (version since last visit), HAMA, HAMD, MoCA scale, QOLIE‐31 scale will be evaluated. Diary cards will be issued, and concomitant medications/non‐drug therapies and adverse events will be recorded. Trial drugs will be dispensed.

Day 31 of administration (V7): Body weight, vital signs, physical examination, blood biochemistry, blood routine, coagulation function, urine routine and ECG will be performed. CGI, PGI, C‐SSRS (since last visit), HAMA, HAMD, MoCA scale and QOLIE‐31 scale will be evaluated. Diary cards will be issued, and concomitant drugs/non‐drug therapies and adverse events will be recorded. Trial drugs will be issued and recovered.

Day 45 of administration (V8): Telephone inquiry about concomitant medications/non‐drug therapies and adverse events will be performed.

On Day 59 of administration (V9): Body weight, vital signs, physical examination, 12 h‐EEG, blood biochemistry, blood routine, coagulation function, urine routine, residual urine volume after urination, OCT examination, ECG examination will be performed. CGI, PGI, C‐SSRS (version since last visit), HAMA, HAMD, MoCA scale, QOLIE‐31 scale will be evaluated. Diary cards will be issued and recovered, and concomitant medications/non‐drug therapies and adverse events will be recorded. Trial drugs will be recovered and PK blood collection will be performed.

#### 2.7.3 Follow‐up period (V10–V11): Discontinuation of drug trial, up to Day 28 post‐discontinuation

On Day 7 post‐discontinuation (V10): Body weight, vital signs, physical examination, blood biochemistry, blood routine, coagulation function, urine routine and ECG will be performed. Diary cards will be issued, and concomitant medications/non‐drug therapies and adverse events will be recorded. PK blood sampling will be performed.

On Day 28 post‐discontinuation (V11): Body weight, vital signs, physical examination, blood biochemistry, blood routine, coagulation function, urine routine, residual urine volume after urination, pregnancy test, OCT test, ECG test will be performed. The diary card will be issued and concomitant medications/non‐drug therapies and adverse events will be recorded.

### 2.8 Sample size calculation

According to the regulations on the number of subjects in the Technical Guidelines for Clinical Pharmacokinetic Studies of Chemical Drugs (2005 Edition), 8 ~ 12 subjects are generally required in each dose group. Therefore, the sample size of this trial is tentatively determined as follows: 3 dose cohorts are preset, 12 subjects (9 investigational drugs and 3 placebo) are planned to be enrolled in each dose cohort, and a total of 36 subjects are planned to be tested.

### 2.9 Randomization

Block randomization is adopted in this trial for each center. The proportion of subjects in the trial group and placebo control group is 3:1. The random number and the treatment group corresponding to the random number are generated by the statistical unit using SAS software. The subjects enter the screening after signing the informed consent form. The naming rules for the screening number of each center are as follows: Site number + S + 2 digits, for example, the first screening number of Site 01 is 01S01, and the second screening number of Site 01 is 01S02. This rule is named according to the screening sequence. Eligible subjects are assigned corresponding random number and drug package number through the randomization system.

Each subject was assigned a randomization number based on the order of enrollment. The randomization numbers were as follows:

Dose Cohort 1:1001–1012.
Dose Cohort 2:2001–2012.
Dose Cohort 3:3001–3012.

Subjects who have been assigned a randomization number and who withdraw or are withdrawn from the clinical trial for any reason, regardless of whether they have taken the investigational product, will retain their randomization number and will not be allowed to re‐enter the trial.

All subjects will be given two numbers, a screening number and a randomization number. Each enrolled subject will first be assigned a screening number, which will be determined by the clinical trial site. Subjects who meet the inclusion criteria will be assigned randomization numbers in ascending order according to the order of enrollment.

The randomization number of the subject will be provided by the statistics department. The experimental group and the control group will be randomly generated at a ratio of 2:1, and the subject will be randomly divided into the experimental group and the control group. Parameters such as the set block length and the initial seed parameters of the random number will be recorded in the randomization table. Subjects who withdraw from the trial early will not be replaced.

### 2.10 Data acquisition

Electronic case report form (eCRF): eCRF was designed and constructed according to the trial protocol. Logic verification was set according to the data verification plan (DVP), and released for use after passing the test by the data administrator and obtaining approval from the sponsor.

Data entry: The eCRF data come from the original records, and the investigator or CRC data entry personnel timely enter the subject visit data into EDC according to the eCRF filling instructions.

Source data on‐site verification (SDV): The monitor checks the consistency of eCRF data and source data, and queries can be issued in case of doubt.

Data queries and answers: Queries originate from systematic queries including EDC logic verification and manual queries by monitors and data managers, and are to be answered in a timely manner by the researcher and/or CRC. Data managers and monitors respond to queries and, if necessary, send queries again until the data are “clean”.

Researcher signature: After data entry and SDV, the researcher will conduct an electronic signature review and confirmation. If there is any data revision after signing, it will need to be re‐signed.

Database lock: Database lock is performed by the data administrator after the database lock record is signed by the lead researcher, sponsor, statistical analyst and data manager.

Database submission: The data manager submits the database to the statistician.

eCRF archiving: For each subject, the eCRF generates a PDF which is stored electronically.

Data Management Report: Written by the data manager.

EDC closure: After the data management report is approved by the sponsor and the data management file is reviewed, the data administrator requests and closes the database.

### 2.11 Research endpoint

Primary endpoint: To assess the safety and tolerability of Pynegabine tablets following multiple oral doses in subjects with focal epilepsy.

Secondary endpoints: (1) to assess the pharmacokinetic (PK) profile of Pynegabine tablets following multiple oral doses in subjects with focal epilepsy and (2) to assess the efficacy of Pynegabine tablets as add‐on treatment for focal seizures, versus placebo.

### 2.12 Statistical analysis

Efficacy analyses of the trial will be performed based on the Full Analysis Set, Safety Set, and PK Set.

### 2.13 Key information

Subject characteristics: Analyses are performed based on FAS, descriptive statistics demographic and other baseline characteristics.

Analysis of medication compliance and concomitant medication: Analysis based on SS, descriptive statistics of drug exposure and exposure time; descriptive statistics of medication compliance, and calculation of compliance ratio of 80%–120%; summary of concomitant medication according to ATC secondary classification and Preferred Name, calculation of number of cases and ratio; detailed description of concomitant medication in tabular form.

### 2.14 Pharmacokinetic analysis

Based on PKS analysis, individual and mean c–t curves were plotted for plasma concentration (c)‐time (t) data analysis. Pharmacokinetic parameters were calculated for each subject by a non‐compartmental model, and arithmetic mean, standard deviation, coefficient of variation, quartile, maximum, minimum and geometric mean of each parameter were calculated for PK parameter analysis. Plasma concentrations before 3 consecutive doses were normalized and linear regression was performed for steady state analysis. Dose proportionality analysis was performed using a power function model (lnPK = *α* + β*lnDose + Error) to analyze the relationship of exposure parameters (Cmax and AUC0–t) to dose.

### 2.15 Validity analysis

Analyses are performed based on FAS, and K‐W H tests are used for descriptive statistics on the percentage change in focal seizure frequency (including the subtypes) per 28 days in the treatment period after 8 weeks of continuous treatment compared with the seizure frequency in the introductory period, the percentage change in focal seizure frequency per week in the treatment period after 1, 2, 3, 4, 5, 6, and 7 weeks of continuous medication compared with the seizure frequency per week in the introductory period, and the change of 12 h‐EEG from baseline after 2 and 8 weeks of continuous treatment.

The chi‐square/Fisher's exact probability method is used to calculate the proportion of subjects with focal seizure frequency (including the subtypes) which reduced by ≥50% per 28 days during the treatment period after 4, 6 and 8 weeks of continuous treatment (response rate), the percentage of subjects without seizure during the treatment period after 2, 4, 6 and 8 weeks of continuous treatment, the proportion of subjects with CGI score ≤2 points after 2, 4 and 8 weeks of continuous treatment, and the proportion of subjects with PGI score ≤2 points after 2, 4 and 8 weeks of continuous treatment.

ANOVA is used for descriptive statistics on the change from baseline in HAMA score after 2, 4 and 8 weeks of continuous treatment, the change from baseline in HAMD score after 2, 4 and 8 weeks of continuous treatment, the change from baseline in MoCA scale score after 2, 4 and 8 weeks of continuous treatment, the change from baseline in QOLIE‐31 scale score after 2, 4 and 8 weeks of continuous treatment, and the change from baseline in NHS3 scale score after 2, 4 and 8 weeks of continuous treatment.

Kaplan–Meier method is adopted to analyze the cumulative time for subjects to reach their average focal seizure frequency per 28 days during the introductory period during the treatment period.

### 2.16 Safety evaluation

Adverse events in the study are classified and assessed according to the National Cancer Institute Common Terminology Criteria for Adverse Events (NCI‐CTCAE v5. 0). The incidence of adverse events is summarized overall and by dose group based on SS safety grade.

## 3 DISCUSSION

In the study of ASMs, due to the diversity of epilepsy causes, genetic heterogeneity and different pathogenesis, no drugs that can fundamentally cure epilepsy have been found at the present stage. The treatment principle of using the least kinds of drugs and the minimum dose to achieve the maximum control of patients’ clinical and subclinical seizures is followed, mainly focusing on 3 objectives: Control seizures, avoid or minimize treatment side effects, and maintain or restore quality of life.^1^

The choice of treatment drug is based on seizure and epilepsy type, epilepsy syndrome, and drug‐related adverse reactions. There are currently more than 25 ASMs approved for the treatment of epilepsy in adults and/or children with different mechanisms of action, including targeting voltage‐gated ion channels (traditional sodium channel blockers such as phenytoin sodium, carbamazepine and oxcarbazepine),^2^, ^3^, ^4^ targeting synapses (Benzodiazepines, barbiturates, Perampanel, levetiracetam, etc.),^5^, ^6^, ^7^, ^8^ acting on multiple targets (valproate, topiramate, lamotrigine, etc.)^9^, ^10^, ^11^ and several other mechanisms. It is not clear which ASM is the most effective or best tolerated, and not all the epileptic patients can achieve seizure‐free. 70% of patients with epilepsy can effectively control their seizures by single drug administration. In patients with difficulty in controlling seizures with single drug administration, only 15–30% can be effectively controlled by combination therapy with different types of ASMs, and up to 80% of patients can be seizure free by using ASMs. Adverse reactions to ASMs are a major cause of reduced quality of life in patients with epilepsy.^12^ Therefore, ASMs with different mechanisms of action are still lacking in clinical practice to further reduce the frequency of seizures, achieve better treatment outcomes and improve patients' quality of life.

Voltage‐gated potassium channels are the most diverse ion channels, encoded by about 40 genes, which are essential for neuronal excitability regulation.^13^, ^14^ To date, multiple epilepsy‐associated potassium channel mutant genes have been identified,^13^, ^15^ but only two drugs targeting potassium channels have been shown to have anti‐seizure effects, namely haloperidol and retigabine, a structural analog of haloperidol, both of which act on Kv7.2/Kv7.3 potassium channels.^16^

To response to the different mechanisms of pharmacological action and adverse reactions of Retigabine, researchers have been trying to find compounds that can stabilize the activity of Kv 7.2/Kv 7.3 and reduce adverse events.^17^ Pynegabine used in this study is a reconfiguration agent of retigabine, a novel potent agonist targeting KCNQ potassium ion channel obtained through multiple rounds of structural optimization and structure–activity studies. Compared with Retigabine, Pyrenegabine exhibits superior stability and increased selectivity towards Kv. It has important clinical value when exploring the safety, tolerance, efficacy and pharmacokinetic characteristics of Pynegabine tablets after multiple oral administration in patients with focal epilepsy.^18^

The emergence of drugs targeting KCNQ ion channels gives clinicians more choices. In the Phase Ia study, the tolerability, safety and pharmacokinetic characteristics of subjects after single oral dose of study drug were evaluated from low to high. The study data showed that Pynegabine tablets had good safety and tolerability, and the risks were controllable. In the Phase Ib study, subjects after oral administration of Pynegabine tablets had good safety and tolerability. At present, there are few multicenter randomized controlled clinical trials of new ASMs, especially class I ASMs, focused on the treatment of Chinese patients with focal epilepsy, and the safety and efficacy of Pynegabine tablets for patients with focal epilepsy still needs further study and evaluation. Therefore, this project intends to conduct a randomized, double‐blind, placebo‐controlled, dose‐escalation clinical trial in collaboration with several epilepsy centers across China to evaluate the safety, tolerability, efficacy and pharmacokinetic profile of Pynegabine tablets for the treatment of patients with focal epilepsy after multiple oral administrations, and to provide more evidence for the clinical application of Pynegabine tablets.

## FUNDING INFORMATION

This study was supported by the National Key R and D Program of China grant (2022YFC2503800), the National Natural Science Foundation of China (82371449, 82201504), Natural Science Foundation of Beijing Municipality (7232045 and Z200024), and Capital Health Research and Development of Special grants (2041–1‐2024), the Beijing Postdoctoral Research Foundation (ZZ 2019‐09), and the Beijing Nova Program (Z211100002121047 and 20220484159).

## CONFLICT OF INTEREST STATEMENT

The authors declare no conflict of interest.

## Data Availability

The data that support the findings of this study are available from the corresponding author upon reasonable request.
